# Supplementary material for: Investigation of the Molecular Epidemiology and Evolution of Circulating Severe Acute Respiratory Syndrome Coronavirus 2 in Thailand from 2020 to 2022 via Next-Generation Sequencing
Source: Viruses. 2023 Jun 19;15(6):1394. doi: 10.3390/v15061394 (PMC10303178; doi:10.3390/v15061394)
Supplement: Supplementary file 1 [file viruses-15-01394-s001.zip › viruses-2469797-supplementary.pdf]

Supplement Table S1. GISAID Accession Numbers.

No	CODE	Accession	Collection date
1	CU5394	EPI_ISL_5880076	11-08-21
2	CU5385	EPI_ISL_5879729	11-08-21
3	CU5614	EPI_ISL_5880433	16-09-21
4	CU5095	EPI_ISL_5879592	30-07-21
5	CU4793	EPI_ISL_5879614	15-07-21
6	CU5615	EPI_ISL_5880434	16-09-21
7	CU5616	EPI_ISL_5880497	16-09-21
8	CU5169	EPI_ISL_5879819	05-08-21
9	CU5378	EPI_ISL_5879908	11-08-21
10	CU5381	EPI_ISL_5879921	11-08-21
11	CU3307	EPI_ISL_4255179	20-05-21
12	CU3313	EPI_ISL_4255418	20-05-21
13	CU5182	EPI_ISL_5879633	05-08-21
14	CU5244	EPI_ISL_5879601	09-08-21
15	CU5608	EPI_ISL_5880288	16-09-21
16	CU3312	EPI_ISL_4255169	20-05-21
17	CU3486	EPI_ISL_5879569	27-05-21
18	CU5174	EPI_ISL_5879597	05-08-21
19	CU5243	EPI_ISL_5879600	09-08-21
20	CU5377	EPI_ISL_5879635	11-08-21
21	CU3585	EPI_ISL_5879605	04-06-21
22	CU5091	EPI_ISL_5879615	30-07-21
23	CU5179	EPI_ISL_5879824	05-08-21
24	CU5171	EPI_ISL_5879823	05-08-21
25	CU5380	EPI_ISL_5879918	11-08-21
26	CU5175	EPI_ISL_5879623	05-08-21
27	CU5384	EPI_ISL_5879923	11-08-21
28	CU618	EPI_ISL_1346630	04-01-21
29	CU3605	EPI_ISL_5879609	04-06-21
30	CU647	EPI_ISL_1346636	22-01-21
31	CU1501	EPI_ISL_4255004	23-03-21
32	CU3394	EPI_ISL_5879603	22-05-21
33	CU617	EPI_ISL_1346628	04-01-21
34	CU632	EPI_ISL_1346631	12-01-21
35	CU646	EPI_ISL_1346634	27-01-21
36	CU1350	EPI_ISL_4254829	09-03-21
37	CU1351	EPI_ISL_4254830	09-03-21
38	CU1208	EPI_ISL_4254674	17-02-21
39	CU1561	EPI_ISL_4254677	3-Jan-21

40	CU1295	EPI_ISL_4254828	03-03-21
41	CU1143	EPI_ISL_4254675	16-02-21
42	CU1475	EPI_ISL_4254726	23-03-21
43	CU1477	EPI_ISL_4254678	23-03-21
44	CU1205	EPI_ISL_4254666	17-02-21
45	CU5176	EPI_ISL_5879626	05-08-21
46	CU5177	EPI_ISL_5879628	05-08-21
47	CU492	EPI_ISL_1346626	21-12-20
48	CU4024	EPI_ISL_5879588	24-06-21
49	CU4028	EPI_ISL_5879589	24-06-21
50	CU5093	EPI_ISL_5879618	30-07-21
51	CU1131	EPI_ISL_4255005	15-02-21
52	CU1398	EPI_ISL_4255166	16-03-21
53	CU4008	EPI_ISL_5879585	24-06-21
54	CU3324	EPI_ISL_4255541	20-05-21
55	CU3687	EPI_ISL_5879581	14-06-21
56	CU3494	EPI_ISL_5879574	27-05-21
57	CU3682	EPI_ISL_5879577	14-06-21
58	CU3062	EPI_ISL_4255167	11-05-21
59	CU3116	EPI_ISL_4255168	14-05-21
60	CU3118	EPI_ISL_4255170	14-05-21
61	CU3135	EPI_ISL_4255171	14-05-21
62	CU3365	EPI_ISL_4255542	22-05-21
63	CU3379	EPI_ISL_4255543	22-05-21
64	CU6392	EPI_ISL_10189523	14-12-21
65	CU6397	EPI_ISL_10189524	16-12-21
66	CU6418	EPI_ISL_10189525	18-12-21
67	CU6994	EPI_ISL_10189526	08-01-22
68	CU6997	EPI_ISL_10189527	08-01-22
69	CU7036	EPI_ISL_10189528	08-01-22
70	CU7112	EPI_ISL_10189532	11-01-22
71	CU7113	EPI_ISL_10189533	11-01-22
72	CU7138	EPI_ISL_10189534	13-01-22
73	CU7141	EPI_ISL_10189536	13-01-22
74	CU7146	EPI_ISL_10189537	13-01-22
75	CU7174	EPI_ISL_10189538	13-01-22
76	CU7176	EPI_ISL_10189544	13-01-22
77	CU7188	EPI_ISL_10238965	13-01-22
78	CU7194	EPI_ISL_10239054	13-01-22
79	CU6743	EPI_ISL_10239055	05-01-22
80	CU6822	EPI_ISL_10239056	05-01-22
81	CU6861	EPI_ISL_10239057	05-01-22

82	CU6866	EPI_ISL_10239119	05-01-22
83	CU6876	EPI_ISL_10239120	05-01-22
84	CU7164	EPI_ISL_10239121	13-01-22
85	CU7167	EPI_ISL_10239155	13-01-22
86	CU7173	EPI_ISL_10239190	13-01-22
87	CU7198	EPI_ISL_10239191	17-01-22
88	CU7354	EPI_ISL_10239276	14-01-22
89	CU7358	EPI_ISL_10239321	14-01-22
90	CU7460	EPI_ISL_10239378	20-01-22
91	CU7461	EPI_ISL_10239438	20-01-22
92	CU7471	EPI_ISL_10239439	20-01-22
93	CU7475	EPI_ISL_10239440	20-01-22
94	CU7476	EPI_ISL_10239505	20-01-22
95	CU7478	EPI_ISL_10239506	20-01-22
96	CU7481	EPI_ISL_10239655	20-01-22
97	CU7526	EPI_ISL_10239656	22-01-22
98	CU7654	EPI_ISL_10239724	27-01-22
99	CU7660	EPI_ISL_10282024	27-01-22
100	CU7659	EPI_ISL_10282086	27-01-22
101	CU7633	EPI_ISL_10282087	27-01-22
102	CU7620	EPI_ISL_10282088	27-01-22
103	CU7618	EPI_ISL_10282089	27-01-22
104	CU7606	EPI_ISL_10282090	26-01-22
105	CU7545	EPI_ISL_10282171	22-01-22
106	CU7655	EPI_ISL_10282172	27-01-22
107	CU7651	EPI_ISL_10282665	27-01-22
108	CU7637	EPI_ISL_10282666	27-01-22
109	CU7547	EPI_ISL_10282744	22-01-22
110	CU7503	EPI_ISL_10282745	21-01-22
111	VOY15	EPI_ISL_11698090	17-01-22
112	VOY17	EPI_ISL_11698091	17-01-22
113	VOY20	EPI_ISL_11698092	17-01-22
114	CU7271	EPI_ISL_11698093	17-01-22
115	CU7546	EPI_ISL_11698094	22-01-22
116	CU7565	EPI_ISL_11698095	22-01-22
117	CU7687	EPI_ISL_11698096	31-01-22
118	CU7700	EPI_ISL_11698097	31-01-22
119	CU7951	EPI_ISL_11698098	10-02-22
120	CU7952	EPI_ISL_11698099	10-02-22
121	CU7957	EPI_ISL_11698100	10-02-22
122	CU8053	EPI_ISL_11698101	14-02-22
123	CU8074	EPI_ISL_11698102	14-02-22

124	CU8140	EPI_ISL_11698103	21-02-22
125	CU8157	EPI_ISL_11698104	22-02-22
126	VOY13	EPI_ISL_11698105	14-01-22
127	CU8229	EPI_ISL_11698106	24-02-22
128	CU8245	EPI_ISL_11698107	24-02-22
129	CU8247	EPI_ISL_11698108	24-02-22
130	CU8265	EPI_ISL_11698109	24-02-22
131	CU8290	EPI_ISL_11698110	24-02-22
132	CU8326	EPI_ISL_11698112	26-02-22
133	CU8356	EPI_ISL_11698114	28-02-22
134	CU8364	EPI_ISL_11711750	28-02-22
135	CU8368	EPI_ISL_11711931	01-03-22
136	CU8374	EPI_ISL_11712097	01-03-22
137	CU8387	EPI_ISL_11713775	01-03-22
138	CU8390	EPI_ISL_11714002	02-03-22
139	CU8394	EPI_ISL_11714167	02-03-22
140	CU8406	EPI_ISL_11714580	02-03-22
141	CU8490	EPI_ISL_12176266	07-03-22
142	CU8492	EPI_ISL_12176267	07-03-22
143	CU8495	EPI_ISL_12176268	07-03-22
144	CU8500	EPI_ISL_12176269	07-03-22
145	CU8503	EPI_ISL_12176324	07-03-22
146	CU8524	EPI_ISL_12387073	07-03-22
147	CU8525	EPI_ISL_12394871	07-03-22
148	CU8530	EPI_ISL_12394872	07-03-22
149	CU8536	EPI_ISL_17770459	08-03-22
150	CU8546	EPI_ISL_17770460	08-03-22
151	CU8762	EPI_ISL_12394973	17-03-22
152	CU8763	EPI_ISL_12394981	17-03-22
153	CU8764	EPI_ISL_17770461	17-03-22
154	CU8765	EPI_ISL_12394983	17-03-22
155	CU8766	EPI_ISL_12394996	17-03-22
156	CU8767	EPI_ISL_12395008	17-03-22
157	CU8768	EPI_ISL_12395018	17-03-22
158	CU8769	EPI_ISL_12395049	17-03-22
159	CU8770	EPI_ISL_17770462	17-03-22
160	CU8771	EPI_ISL_17770463	17-03-22
161	CU8828	EPI_ISL_17770465	21-03-22
162	CU8907	EPI_ISL_17770466	24-03-22
163	CU8936	EPI_ISL_17770467	25-03-22
164	CU8941	EPI_ISL_17770468	25-03-22
165	CU8986	EPI_ISL_17776299	04-04-22

166	CU9023	EPI_ISL_17776603	04-04-22
167	CU9027	EPI_ISL_17776604	04-04-22
168	CU9034	EPI_ISL_17776605	04-04-22
169	CU9045	EPI_ISL_17778770	04-04-22
170	CU9058	EPI_ISL_17778771	04-04-22
171	CU9059	EPI_ISL_17779272	04-04-22
172	CU9049	EPI_ISL_17779273	04-04-22
173	CU9263	EPI_ISL_17779913	26-04-22
174	CU9331	EPI_ISL_17779914	11-05-22
175	CU9354	EPI_ISL_17779915	11-05-22
176	CU9355	EPI_ISL_17779916	11-05-22
177	CU9357	EPI_ISL_17779940	11-05-22
178	CU9360	EPI_ISL_17779941	11-05-22
179	CU9363	EPI_ISL_17779942	11-05-22
180	CU9366	EPI_ISL_17779943	11-05-22
181	CU9371	EPI_ISL_17779944	11-05-22
182	CU9394	EPI_ISL_17779948	11-05-22
183	CU9401	EPI_ISL_17779949	11-05-22
184	CU9408	EPI_ISL_17779950	11-05-22
185	CU9413	EPI_ISL_17779951	11-05-22
186	CU9436	EPI_ISL_17779952	11-05-22
187	CU9437	EPI_ISL_17779953	11-05-22
188	CU9662	EPI_ISL_17779954	17-06-22
189	CU9679	EPI_ISL_17779955	17-06-22
190	CU9684	EPI_ISL_17779956	17-06-22
191	CU9689	EPI_ISL_17779957	17-06-22
192	CU9693	EPI_ISL_17779958	17-06-22
193	CU9700	EPI_ISL_17779959	17-06-22
194	CU9709	EPI_ISL_17779960	17-06-22
195	CU9713	EPI_ISL_17779961	20-06-22
196	CU9714	EPI_ISL_17779962	20-06-22
197	CU9722	EPI_ISL_17779963	20-06-22
198	CU9723	EPI_ISL_17779964	20-06-22
199	CU9726	EPI_ISL_17779965	20-06-22
200	CU9728	EPI_ISL_17779966	20-06-22
201	CU9730	EPI_ISL_17779967	20-06-22
202	CU9737	EPI_ISL_17779968	20-06-22
203	CU9778	EPI_ISL_17779969	27-06-22
204	CU9779	EPI_ISL_17779970	27-06-22
205	CU9816	EPI_ISL_17779971	04-07-22
206	CU9819	EPI_ISL_17779972	04-07-22
207	CU9822	EPI_ISL_17779973	04-07-22

208	CU9829	EPI_ISL_17779974	05-07-22
209	CU9836	EPI_ISL_17779975	06-07-22
210	CU9842	EPI_ISL_17779976	06-07-22
